# Is three-dimensional–printed custom-made ultra-short stem with a porous structure an acceptable reconstructive alternative in peri-knee metaphysis for the tumorous bone defect?

**DOI:** 10.1186/s12957-021-02355-7

**Published:** 2021-08-08

**Authors:** Jie Wang, Jingjing An, Minxun Lu, Yuqi Zhang, Jingqi Lin, Yi Luo, Yong Zhou, Li Min, Chongqi Tu

**Affiliations:** 1 Department of Orthopaedics, Orthopaedic Research Institute, West China Hospital, Sichuan University, Chengdu, People’s Republic of China; 2 Bone and Joint 3D-Printing and Biomechanical Laboratory, Department of Orthopaedics, West China Hospital, Sichuan University, Chengdu, People’s Republic of China; 3 Department of Operating Room, West China Hospital, Sichuan University/ West China School of Nursing, Sichuan University, Chengdu, People’s Republic of China

**Keywords:** Three-dimensional-printed, Custom-made, Stem, Porous structure, Peri-knee metaphysis

## Abstract

**Background:**

Long-lasting reconstruction after extensive resection involving peri-knee metaphysis is a challenging problem in orthopedic oncology. Various reconstruction methods have been proposed, but they are characterized by a high complication rate. The purposes of this study were to (1) assess osseointegration at the bone implant interface and correlated incidence of aseptic loosening; (2) identify complications including infection, endoprosthesis fracture, periprosthetic fracture, leg length discrepancy, and wound healing problem in this case series; and (3) evaluate the short-term function of the patient who received this personalized reconstruction system.

**Methods:**

Between September 2016 and June 2018, our center treated 15 patients with malignancies arising in the femur or tibia shaft using endoprosthesis with a 3D-printed custom-made stem. Osseointegration and aseptic loosening were assessed with digital tomosynthesis. Complications were recorded by reviewing the patients’ records. The function was evaluated with the 1993 version of the Musculoskeletal Tumor Society (MSTS-93) score at a median of 42 (range, 34 to 54) months after reconstruction.

**Results:**

One patient who experienced early aseptic loosening was managed with immobilization and bisphosphonates infusion. All implants were well osseointegrated at the final follow-up examination. There are two periprosthetic fractures intraoperatively. The wire was applied to assist fixation, and the fracture healed at the latest follow-up. Two patients experienced significant leg length discrepancies. The median MSTS-93 score was 26 (range, 23 to 30).

**Conclusions:**

A 3D-printed custom-made ultra-short stem with a porous structure provides acceptable early outcomes in patients who received peri-knee metaphyseal reconstruction. With detailed preoperative design and precise intraoperative techniques, the reasonable initial stability benefits osseointegration to osteoconductive porous titanium, and therefore ensures short- and possibly long-term durability. Personalized adaptive endoprosthesis, careful intraoperative operation, and strict follow-up management enable effective prevention and treatment of complications. The functional results in our series were acceptable thanks to reliable fixation in the bone-endoprosthesis interface and an individualized rehabilitation program. These positive results indicate this device series can be a feasible alternative for critical bone defect reconstruction. Nevertheless, longer follow-up is required to determine whether this technique is superior to other forms of fixation.

## Background

Nowadays, advancing radiography techniques, progressing adjuvant therapies, and precise preoperative simulation enable surgeons to perform challenging limb-salvage surgeries to restore normal joints, especially around the knee [1–5]. These joint-preserving procedures usually result in critical bone stumps with a length ranging from 4 to 10 cm, with which effective fixation is hard to obtain owing to reverse funnel-shaped anatomy, enlarged sectional diameter and reduced anchorage length of the peri-knee metaphysis [6, 7].

Previously, numerous methods including autografts [8–11], allografts [12–15], autograft–allograft composite [16–18], induced membrane technique [19, 20], distraction osteogenesis [21–23] and metallic components [13, 24, 25] have been proposed. Biologic reconstructions can provide bioactive bone tissue once ideal healing is achieved. However, these procedures are time-consuming and accompanied by a high incidence of complications, such as infection, delayed union, nonunion, and fracture [8, 12–19, 26–28]. Additionally, the application of biological reconstruction can be limited if either side of the articular surface is involved. Therefore, the intercalary endoprosthesis is favored by some surgeons to substitute the massive bone shaft, because of its advantages including rigid initial stability, adjustable length for diversified bone defects, and relatively rapid restoration of function [29, 30]. Nevertheless, endoprostheses are prone to mechanical complications around the bone implant interface constructs, such as aseptic loosening, endoprosthetic fracture, and periprosthetic fracture [29–32].

The establishment method of bone implant interface construct, including a compressive osseointegration method, a cemented stem, or an uncemented stem, is crucial for the performance of each intercalary endoprosthesis [13, 24, 33]. The compressive osseointegration method has been documented with reasonable 5-year and 10-year survival by enhancing osseointegration, yet its application in critical bone defects around the knee is rare [34, 35]. The cemented stem contributes to rigid early fixation but lacks osteoconductivity, resulting in widely reported aseptic loosening (range, 0 to 53.8%) [29, 31, 32, 35–38]. Besides, cross-pin has been introduced to cemented fixation in the peri-knee metaphysis, whereas the strength of bone-stem construct is reduced by the centric pinholes, leading to foreseeable stem fracture [30]. The uncemented stem in the literature, with the ability to facilitate osseointegration, is rarely reported because inadequate bone stock of peri-knee metaphysis can fail to provide sufficient initial stability [6, 32, 39]. Hence, an alternative stem with improved adaptation to the stump, enhanced mechanical strength, reinforced primary stability and predictable osseointegration is expected. With the rapid progress in additive manufacturing techniques, three-dimensional (3D)–printed custom-made endoprosthetic stem with a porous structure provides an option for such ultra-short and dilated medullary cavity.

Our previous study has reported the application of 3D-printed intercalary endoprosthesis in tibial ultra-critical bone defect with a bone stump under 4-cm length [40]. In this study, we designed a series of 3D-printed custom-made ultra-short stems with a porous structure for peri-knee metaphysis with a bone stump ranging from 4- to 10-cm length. We wish to determine, at a minimum of 2 years, the early performance of this new device in the peri-knee metaphysis. The purposes of this study were to (1) assess osseointegration between host bone and 3D-printed custom-made stem with a porous structure and correlated incidence of aseptic loosening; (2) identify complications including infection, endoprosthesis fracture, periprosthetic fracture, and wound healing problem in all patients, and leg length discrepancy in skeletally immature patients; and (3) evaluate the short-term function of the patient who received this personalized reconstruction system.

## Methods

### Patients

Between September 2016 and June 2018, our center treated 15 patients with malignancies arising in the femur or tibia. The indications were (1) tumors with no evidence of progression clinically or on magnetic resonance imaging (MRI) studies during chemotherapy and (2) following en-bloc resection, the short residual bone stump length ranging from 4 to 10 cm, impossible for standard uncemented stem fixation in our institution. The contraindications were (1) not willing to accept the potential risks of the 3D-printed custom-made endoprosthesis, and (2) the bone stump with a minimum medulla sectional diameter under 9 mm. There were six men and nine women with a median age of 22 years (range, 11 to 61 years) (Table 1).

**Table 1:** The demographics of the fifteen patients who received reconstruction with endoprosthesis with three-dimensional–printed custom-made ultra-short porous stem in peri-knee metaphysis

	Age (year)	Gender	Diagnosis	Tumor location	Enneking staging [41]	Neo-adjuvant chemotherapy	Follow-up (month)	Oncologic outcome	Complications	MSTS-93 score	Knee ROM (°)
Patient											
1	27	F	Ewing sarcoma	Femur, D	IIB	Two circles	42	NED	PPF	30	140
2	61	M	Chondrosarcoma	Femur, P	IIB	No	39	NED		25	110
3	50	M	Chondrosarcoma	Femur, P	IIB	No	50	NED		24	100
4	11	F	Osteosarcoma	Femur, D	IIB	Two circles	48	NED		27	130
5	57	F	MLC	Femur, D	NA	No	36	AWD		27	120
6	30	F	Osteosarcoma	Femur, P	IIB	Two circles	43	NED		24	120
7	14	M	Osteosarcoma	Femur, P	IIB	Two circles	45	NED		25	110
8	45	M	POS	Femur, D	IIB	No	40	NED		28	120
9	13	M	Osteosarcoma	Tibia, D	IIB	Two circles	41	NED	PPF+EAL	29	120
10	19	F	Ewing sarcoma	Tibia, D	IIB	Two circles	34	NED		30	140
11	22	F	Osteosarcoma	Tibia, D	IIB	Two circles	54	NED		23	110
12	38	F	Osteosarcoma	Tibia, D	IIB	Two circles	48	NED		28	130
13	16	F	Osteosarcoma	Tibia, D	IIB	Two circles	37	NED		26	120
14	12	M	Ewing sarcoma	Tibia, D	IIB	Two circles	43	NED		26	120
15	15	F	Osteosarcoma	Tibia, D	IIB	Two circles	42	NED		25	110
Median	22						42			26	120

*MLC*, metastatic lung cancer; *POS*, periosteal osteosarcoma; *P*, proximal; *D*, diaphysis; *NA*, not applicable; *NED*, no evidence of disease; *AWD*, alive with disease; *PPF*, periprosthetic fracture; *EAL*, early aseptic loosening; *MSTS*, musculoskeletal tumor society; *ROM*, range of motion.

Diagnoses were osteosarcoma in eight patients, Ewing sarcoma in three, chondrosarcoma in two, parosteal osteosarcoma in one, and metastatic lung cancer in one. The tumor locations were eight femora (four proximal, four diaphyseal) and seven tibia diaphysis. According to the Enneking staging system [41], 14 patients with osteosarcoma, Ewing sarcoma, chondrosarcoma, and parosteal osteosarcoma had Stage IIB disease. Among the 11 patients in the study, two cycles of neoadjuvant chemotherapy were administered in eight patients with osteosarcoma (doxorubicin and cisplatin) and three with Ewing sarcoma (vincristine, doxorubicin, cyclophosphamide/ifosfamide, and etoposide) (Table 1).

**Fig. 1:**
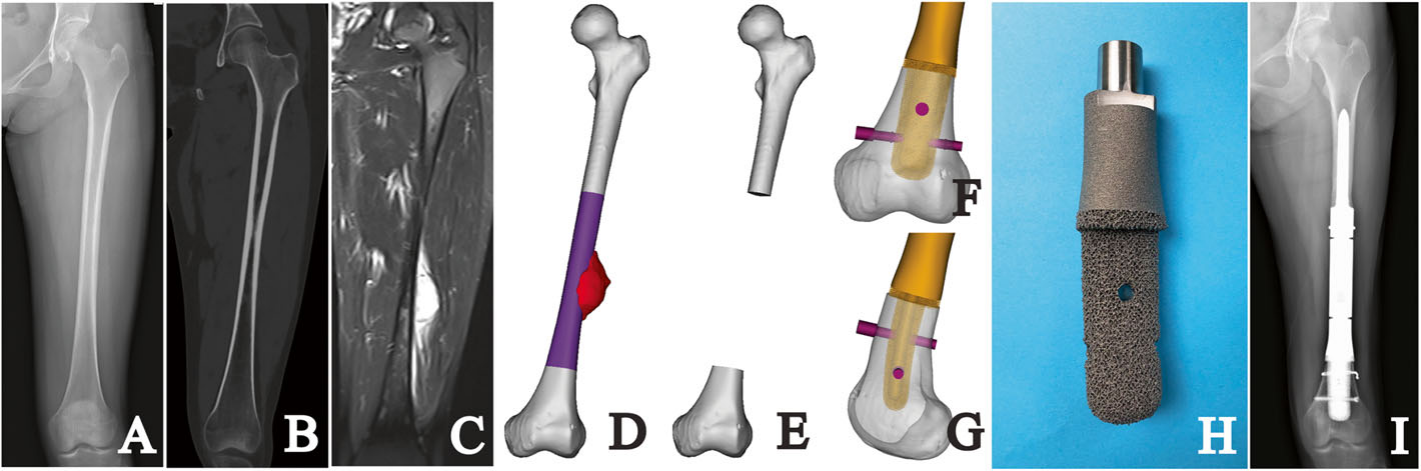
**A** The AP radiographic view shows an Ewing sarcoma of the diaphyseal left femur (patient 1). The **B** CT and **C** MR images show extension in the surrounding tissues. The virtual **D** tumor model and **E** defect model in Mimics software is shown. The **F** AP view and **G** lateral view of stem design in distal femoral stump are shown. **H** The photograph shows the porous structure and whole 3D-printed custom-made stem fabricated with electronic beam melting technique. **I** The AP radiograph at the latest follow-up is shown

Preoperatively, all patients underwent plain radiography, 3D-computerized tomography (CT), and MRI of their lesions. Single-photon emission CT, chest CT, and biopsy were also performed (Fig. 1A–C).

This study was approved by the ethical committee of our institution. Written informed consent to participate in this study was obtained from all patients.

### Endoprosthesis design and fabrication

All endoprostheses were designed by our clinical team and fabricated by Chunli Co., Ltd. (Tongzhou, Beijing, China). 3D-CT and MRI data were integrated by the image fusion technique to build virtual bone and tumor models in Mimics V20.0 software (Materialise Corp., Leuven, Belgium) (Fig. 1D). After confirming the tumor margin, virtual osteotomy was undertaken with an individualized tumor-free bone resection margin (Fig. 1E). The cross-sectional diameters of the maximum inscribed circle at the osteotomy point were measured to assess whether a custom-made stem was required. If so, on the cross-sectional plane, a maximum-inscribed ellipse whose long axis was parallel to the coronal plane was then set and measured as design data of the stem base. To determine the stem length, the depth of the medullary cavity of the stump was measured from the osteotomy point to the endpoint, which varied in skeletally mature and immature patients. In skeletally mature patients, the endpoint was set as the intercondylar notch in the femur and articular surface in the tibia. While in skeletally immature patients, the endpoint was set as the epiphyseal plate. The design of stem length preserved some depth for possibly aseptic loosening, and avoided the interruption to the epiphyseal plate in skeletally immature patients. Before generating the stem according to the size of the previous ellipse and the depth of the preserved stump (Fig. 1F,G), the medullary cavity of the stump was evaluated to determine whether a press-fit fixation can be applied. If a press-fit fixation was accessible, the bone condition would be assessed basically in line with patients’ age. For the patients over 12 years old, their bone condition is usually considered mature enough to enable a press-fit fixation. For the patients under 12 years old, their bone condition including secondary sexual characteristics, height, and body weight would be comprehensively evaluated. Additionally, the first menstruation was an important reference for girl patients. If the bone condition was considered sufficient, a press-fit fixation would be utilized; otherwise, a sub-press-fit fixation using a 1-mm-smaller stem would be applied. After stem tapering, a 2.5-mm-thick porous layer was split to cover the inside solid stem. Cross-pins of 5-mm diameter, with the direction of anteroposterior or transversal, went through the inside solid stem eccentrically to ensure fixation durability. The anteroposterior cross-pins were carefully located if the osteotomy point was near the patellofemoral articular surface in the femur or tuberosity of the tibia. With a porous layer, the contact surface of the stem shoulder matched the outline of the cross-section at the osteotomy point. Meanwhile, two drilling indicators were designed on the extracortical portion of the endoprosthesis stem to guide cross-pin alignment. Thereafter, the following two models were designed to assist implantation. The orientation model had the same stem shoulder and diameter as the endoprosthesis, whereas only 2-mm length of the stem was kept with a sharp edge. The reamer-indicating model had the same stem shoulder and 1-mm thinner stem compared with the real endoprosthesis.

The median length of the resected segment, depth of the preserved medullary cavity, stem, longer and shorter axes of stem section, and length proportion of resected segment to total length were 218.7 mm, 64.4 mm, 51.2 mm, 23.7 mm, 15.8 mm, and 57.4%, respectively. The median number of cross-pins designed in peri-knee metaphysis was 2 (range, 0 to 2) (Table 2).

**Table 2:** The detailed information of measured and design data

	Resected length (mm)	Depth of preserved medullary cavity (mm)	Length proportion of resected to total (%)	Stem length (mm)	Longer axis length of stem section (mm)	Shorter axis length of stem section (mm)	Number of cross-pins	Leg length discrepancy (mm)
Patient								
1	170	77.5	42.7	61.4	25.8	15.8	2	2
2	314.7	46.8	87.3	36.5	33.7	21.8	1	2
3	333.4	90.9	76.8	76.6	23.7	16.1	2	1
4	146.2	43.4	48.7	35.2	23.6	15.1	2	2
5	167.9	87.4	50.9	65.3	19.6	15.8	2	0
6	301.6	97.1	75.6	80.8	18.5	14.2	2	1
7	323.2	92.7	77.7	80	20.2	15.3	2	13
8	188.7	62.7	47.3	51.2	26.8	17.9	2	0
9	233.1	84.4	68.5	47	16.3	15.3	0	12
10	287.3	58.4	79.7	47.8	27.2	23.2	2	1
11	191.8	60.3	64.6	44.1	20.8	15.4	2	0
12	218.7	68.5	57.4	56.6	23.9	17.5	2	0
13	187.4	44.1	51.6	36.1	26.7	18.5	2	2
14	220.4	60.3	54.6	40.2	27	18.6	2	1
15	162.1	64.4	50.3	53.2	19.7	15.4	2	1
Median	218.7	64.4	57.4	51.2	23.7	15.8	2	1

The endoprosthesis was fabricated with Ti6Al4V alloy using the electron beam melting technique (ARCAM Q10plus, Mol̈ndal, Sweden) (Fig. 1H); meanwhile, the patient-specific instruments and endoprosthesis models were fabricated using the stereolithography appearance technique (UnionTech Lite 450HD, Shanghai, China). The workflow of design, fabrication, and delivery costs about 10 days.

### Surgical techniques

All surgeries were performed by the same senior surgeon. Standard techniques for tumor exposure were used, with the principle of obtaining a wide surgical margin. Thereafter, the osteotomy was undertaken precisely with the aid of patient-specific instruments. The orientation model was attached to the host bone tightly to obtain an ellipse mark for the following reaming. The medullary cavity was then reamed with bone files precisely and the trabecular bone was gathered for later autografting. In skeletally immature patients, the medullary cavity was usually un-reamed. After checking the interfacial fitness between the host bone and the orientation model, the trial implantation with the reamer-indicating model was undertaken to ensure whether reaming was adequate or not. The following step was the implantation of the endoprosthesis stem. In skeletally immature patients who were expected to receive a press-fit fixation, the stump was tied using a wire in advance. After the insertion, the stump was fixed with wires before reinserting the stem in all patients who encountered an intraoperative periprosthetic fracture. The cortex was drilled according to the drilling indicators before inserting the cross-pins. Afterward, the reduction of all modular segments (40 to 120 mm, Chunli Co., Ltd., Tongzhou, Beijing, China) of different lengths was undertaken according to the preoperative plan. At last, the muscles and soft tissue were sutured tightly layer by layer. Meanwhile, in the proximal tibia, rotation of gastrocnemius myocutaneous flap and free skin grafting were performed when necessary to ensure soft tissue coverage [40].

### Postoperative management

The rehabilitation protocol was personalized according to tumor location, the length of the resected regimen and preserved host bone, the size of the stem, and the bone condition of the patient. For the patient who received a tibial intercalary endoprosthetic replacement, muscle training was undertaken to reinforce the strength and balance of the lower limb during the first 4 weeks. From 4 weeks postoperatively, standing without weight-bearing was allowed for 2 weeks. Thereafter, patients were encouraged to gradually increase weight-bearing on the affected limb from 10 kg until weight-bearing was equal to that of the contralateral side, and this process usually lasted for 2 weeks. Further, single-leg standing on affected limb and ambulation with walking aids were the next process in the following 4 weeks. Squat training was the last program and usually began 3 months after the surgery.

For the patient who received a femoral intercalary endoprosthetic replacement, the rehabilitation schedule was tighter comparing to former group patients. The muscle training was undertaken in the first 2 weeks after the surgery and followed by a non-weight-bearing stance for 2 weeks. Increasing–weight-bearing stance began at 4 weeks postoperatively and lasted for 2 weeks. Afterward, single-leg stance and ambulation with walking aids for 2 weeks were arranged. At last, squat training was suggested at 2 months postoperatively.

For the patient who received a proximal femoral replacement and hip hemiarthroplasty, the rehabilitation program was similar to patients who received hemipelvic replacement [42]. The total rehabilitation period cost about 3 months.

**Fig. 2:**
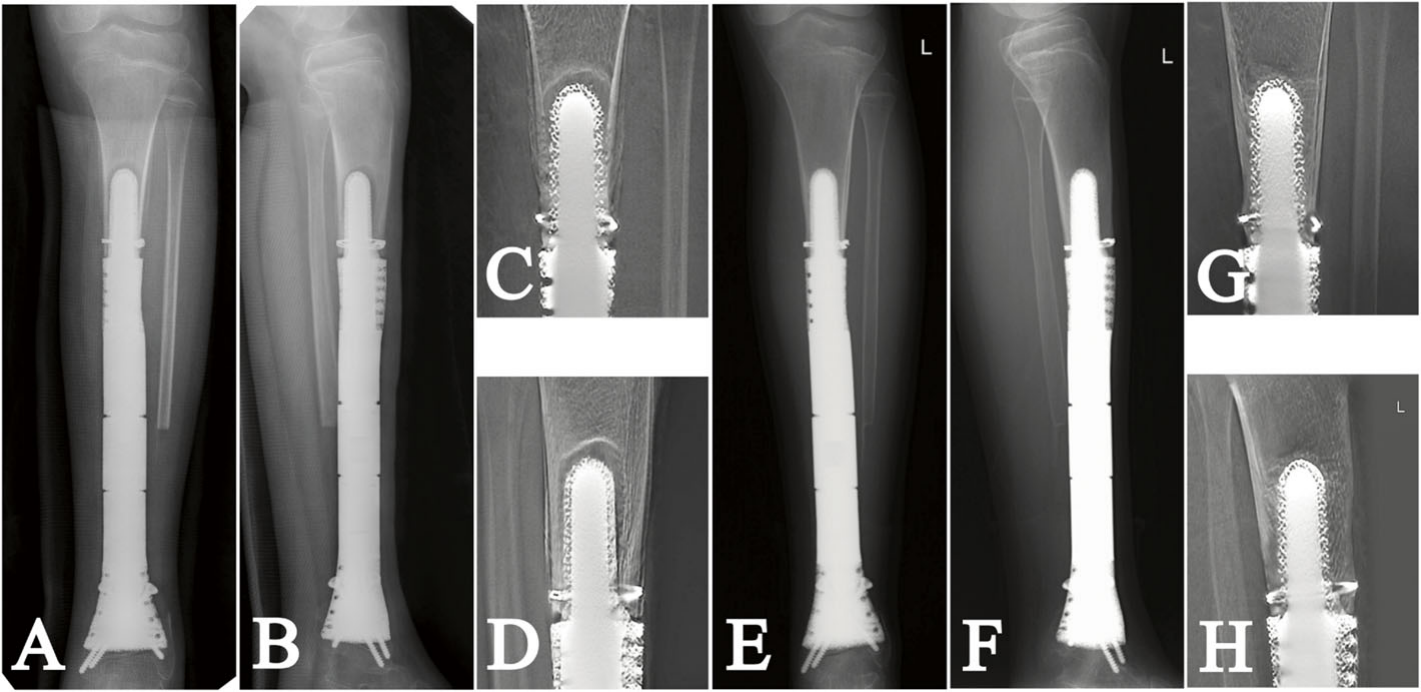
The **A** AP and **B** lateral plain radiographs show early aseptic loosening in patient 9 3 months postoperatively; The **C** AP and **D** lateral digital tomosynthesis graphs show radiolucency and hardened area around the stem. The **E** AP and **F** lateral plain radiographs and **G** AP and **H** lateral digital tomosynthesis images show well osseointegration at latest follow-up

All patients were evaluated with a physical examination, plain radiography of the femur or tibia before discharge, and once a month during the first 3 months postoperatively and quarterly thereafter (Fig. 1I). Meanwhile, we used digital tomosynthesis (Sonialvision Safire II, Shimadzu, Kyoto, Japan) to assess osseointegration at the bone implant interface (Fig. 2). At a median follow-up interval of 42 months (range, 34 to 54 months), no patient was lost to follow-up, 14 patients had no evidence of disease, and one patient with metastatic lung cancer was alive with disease (Table 1).

### Primary and secondary study endpoints

Our primary endpoint of interest was osseointegration. Digital tomosynthesis was performed every 3 months postoperatively. Two senior surgeons independently evaluated digital tomosynthesis images at bone implant interfaces. We observed the trabecular structures connected to the implant surface to assess whether there was good osseointegration [43–45]. While the aseptic loosening was defined as radiolucency between stem and hardened trabecular bone.

Our second endpoint was complications. Complications including deep infection, wound healing problem, periprosthetic fracture, endoprosthesis fracture, local recurrence, and metastasis were recorded. The leg length discrepancy was measured using a full-length lower limb radiograph at latest follow-up.

Our third endpoint was function. The MSTS-93 score was assessed through a review of patient records undertaken by a surgeon who was not involved in the patient’s care [46]. The MSTS-93 is a limb-specific assessment based on six categories (pain, function, emotional acceptance, supports, walking ability, and gait) specific to the entire lower limb. Each category is scored from 0 to 5, with a total score from 0 to 30 (a higher score is desirable). The MSTS-93 score was administered at the most recent follow-up examination. Meanwhile, the range of motion of the knee joint was measured.

## Results

### Osseointegration

**Fig. 3:**
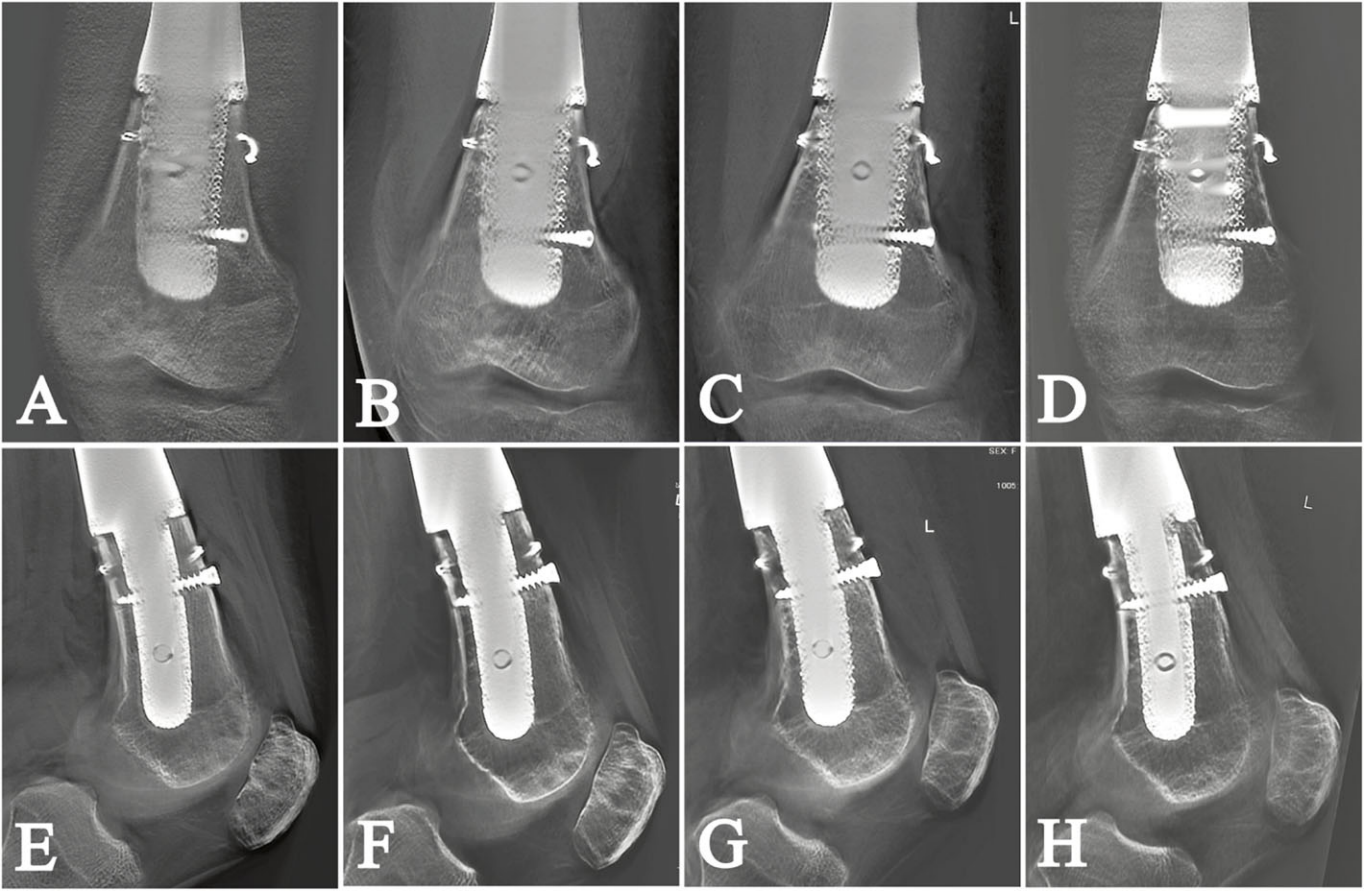
The postoperative AP digital tomosynthesis graphs (patient 1) at **A** 3, **B** 6, **C** 12, and **D** 24 months show well osseointegration. The postoperative lateral digital tomosynthesis graphs at **E** 3, **F** 6, **G** 12, and **H** 24 months are shown

One patient encountered early aseptic loosening and severe osteoporosis 3 months postoperatively (Fig. 3). The patient’s lower limb was immobilized by plaster, and weight-bearing was forbidden for 1 month, and bisphosphonate was applied thereafter. All implants were well-osseointegrated at the final follow-up examination (Table 1).

### Complications

There are two periprosthetic fractures intraoperatively. A wire was applied to assist fixation, and the fracture healed at the latest follow-up. No deep infection, wound healing problem, endoprosthesis fracture, local recurrence, or metastasis was observed during the follow-up (Table 1). The median leg length discrepancy was 1 mm (range, 0 to 13 mm). Among six skeletally immature patients, two patients experienced significant leg length discrepancy of 12 mm (patient 7) and 13 mm (patient 9) (Table 2).

### Function

The median MSTS-93 score of all patients was 26 (range, 23 to 30). Specifically, the median MSTS-93 scores of patients who received femoral intercalary endoprosthesis replacement, tibial intercalary endoprosthesis replacement, and proximal femoral endoprosthesis replacement and hip hemiarthroplasty were 27.5 (range, 27 to 30), 26 (range, 23 to 30), and 24.5 (range, 24 to 25), respectively. The median range of motion of the knee joint was 120° (range, 100 to 140°) (Table 1).

## Discussion

Endoprostheses provided a viable alternative to biological methods for the massive bone defect in the lower limb because their immediate stability restores reasonable support for early rehabilitation and enables rapid recovery of function [1, 29–33, 36–38, 47, 48]. However, in some severe situations with a shortened residual segment, further application of this device is limited due to the incidence of mechanical failures [7, 29, 30, 32, 33, 36, 38, 47–49] (Table 3). A novel 3D-printed custom-made stem with a porous structure might minimize the occurrence of mechanical complications. We found satisfactory osseointegration was obtained, and there was a low incidence of complications with acceptable function following the utilization of such a device in this series.

**Table 3:** Detailed data of previous fixation with stem in peri-knee metaphysis

Study	Patient number	Stump location	Fixation tech	Manufacturer	Stump length (cm)	Stem length (cm)	Mean follow-up (months)	MSTS-93 score	Complications
Guder 2017 [31]	4	4 Tibiae	Rough, hollow stem; uncemented	Implantcast, Buxtehude, Germany	3.1 (2.5–3.5)	-	56 (30–102)	28 (27–29)	2 Wound healing, 1 local recurrence, 2 periprosthetic fracture
Burger 2016 [7]	5	5 Femora	Spreading stem; uncemented	ArgoMedical, Zug, Switzerland	> 12	12	21.5 (3.5–46)	19 (7–26)	1 Metastasis
Bernthal 2019 [30]	56	12 Femora, 1 tibia, 43 others	Cross-pin; cemented	Stryker, Mahwah, New Jersey)	-	-	Median 132 (IQR, 44–189)	-	1 Infection, 2 aseptic loosening, 1 structural failure
Benevenia 2016 [29]	41	21 Femora, 5 tibiae, 16 humeri	Clamped intramedullary-nail, cemented /uncemented	OsteoBridge IDSF; Merete, Berlin, Germany	-	1–20	14 (1–51)	Femur 25 (10–29), tibia 23 (13–30)	5 Aseptic loosening (uncemented), 6 structural failure (cemented)
Aldyami 2005 [25]	35	29 Femora, 3 tibiae, 3 humeri	Cemented /uncemented	-	-	-	107 (24–306)	-	14 Metastasis, 5 local recurrences, 1 periprosthetic fracture, 2 prosthetic fracture, 7 aseptic loosening
Hanna 2010 [36]	23	23 Femora	Fluted stem and extracortical plates, cemented, and HA collar	Stanmore Implants Worldwide Ltd, Middlesex, UK	-	-	97 (3–240)	26 (20–28)	1 Infection, 2 prosthetic fracture, 1 periprosthetic fracture, 1 aseptic loosening
Sewell 2011 [38]	18	18 Tibiae	Fluted stem, cemented, and HA collar	Stanmore Implants Worldwide Ltd, Middlesex, UK	-	2.8–7	58.5 (20–141)	23 (17–28)	4 Aseptic loosening (proximal), 1 infection, 2 periprosthetic fracture
Stevenson 2017 [50]	37	9 Femora, 28 others	Extracortical plates, cemented, and HA collar	Stanmore Implants Worldwide Ltd, Middlesex, UK	-	7.9 (3.4–10)	84 (12–204)	-	2 Aseptic loosening, 1 structural failure, 1 infection
Streitburger 2020 [32]	28	17 Femora, 11 tibiae	Solid or hollow, hexagonal stem with protruding fins and interlocking screw	Implantcast, Buxtehude, Germany	2.5–26	-	35 (4–139)	-	14 Aseptic loosening (4 femora, 4 tibiae), 1 periprosthetic fracture, 3 infection, 4 local recurrence
Tedesco 2017 [35]	6	5 Femora, 1 tibia	Double compressive osseointegration	Biomet, Inc, Warsaw, Indiana	3.7–8.2	-	39 (10–108)	26 (11–30)	2 Prosthetic fracture, 1 periprosthetic fracture
Current study	15	8 Femora, 7 tibiae	3D-printed stem with a porous structure	Chunli Co., Ltd. Tongzhou, Beijing, China	Median 6.4 (44.1–97.1)	Median 5.1 (42.7–79.7)	Median 42 (34–54)	Median 26 (23–30)	2 Periprosthetic fracture, 1 early aseptic loosening

There were several limitations and biases in this study. First, we did not evaluate the oncologic outcome because the small cohort of 15 patients and diverse diagnoses were considered inadequate to assess this outcome. Second, we included both femoral and tibial replacements to obtain a general understanding of this reconstruction method in the lower extremity. The included patients varied in age, height, weight, and treatment protocol. Therefore, one should be careful when introducing our results to individual patients. Third, surgeons’ subjective assessment of osseointegration might result in assessment bias. To mitigate this, the radiography was assessed independently by two surgeons, and clinical outcomes such as pain relief and improved ambulation were also analyzed [42]. Hence, the influence of assessment bias was not severe. Finally, with a short follow-up duration and a small series of 15 patients, the drawbacks might be hindered. Therefore, larger multicenter studies with prolonged follow-up period are needed to evaluate this approach.

All implants were well osseointegrated at the final follow-up examination. Previously, with the wide application of cement in the cemented reconstruction of critical bone defects, the initial stability was relatively easy to secure with the assistance of extracortical plate or cross-pins [29, 30, 33, 36, 38, 47, 48]. While in uncemented fixation, it is challenging to ensure adequate initial stability of the stem in a critical stump due to the absence of cement [1, 32]. Several attempts including spreading stem, hollow stem, and compressive osseointegration fixation have been introduced, while they are limited by the extended length of spreading stem, high aseptic loosening rate of the hollow stem, and common aseptic loosening and endoprosthesis fracture of compressive osseointegration fixation [7, 32, 49]. Additionally, fixation with extracortical plate seems a viable stability booster, whereas it requires adequate cortex exposure which might imperil the attachment of peri-knee non-osseous structures, and therefore impair joint stability and healing process. Hence, the uncemented stem in our series was modified in the following aspects to strengthen both initial stability and osteoconductivity. First, the stem has an ellipse cross-section, utilizing most of the remaining medullary cavity to provide adequate anti-rotation force. Second, the curved stem in the femur and straight stem in tibia sticking to posterior cortex match weight transmission and ensure anti-rotation property. Third, the cross-pins throughout cortex and stem provide extra resistance to rotation and forward or backward leaning. Fourth, before inserting the stem, the medullary cavity was reamed to accommodate the stem model, which was thinner than the real stem for 1 mm. This procedure provides a tight connection to defend the rotation and lean of the stem. Finally, the stem integrated a 2.5-mm-thick porous structure with 600-μm pore size and 60% porosity rather than coating surface, to facilitate osseointegration [51–55]. In our series, one early aseptic loosening occurred in a 13-year-old patient with a stem under 5-cm length. The cross-pin was absent to prevent endoprosthesis fracture, considering thinner stem in his relatively small medullary cavity might be weakened in strength by pinholes. Additionally, a shorter stem was determined because the residual diaphyseal cortex was expected to provide partial press-fit fixation. Intraoperative stability test indicated positive results; nevertheless, stem instability and severe osteoporosis were observed with poor osseointegration at 3 months postoperatively. Thereafter, 1-month immobilization and bisphosphonate were administrated, contributing to final satisfactory osseointegration. Hence, for the stems with a porous structure under 5 cm, osseointegration is expectable with adequate cross-pins, proper immobilization, and probable bisphosphonate [56].

Besides aseptic loosening, endoprosthesis reconstructions involving peri-knee metaphysis are associated with complications including periprosthetic fracture and endoprosthesis fracture [29–36, 38, 47, 48, 50]. In our series, two intraoperative periprosthetic fractures occurred. The mild crack fracture might result from the following two reasons. First, owing to the curving design of the stem, implanting the stem requires a larger section when the midpoint of the stem is inserting into the medullary cavity. Additionally, the cortex near metaphysis is thinner than the diaphyseal cortex. After the fixation with wire and cross-pins, the initial stability was considered adequate, and the fracture healed during the follow-up. In the literature, periprosthetic fractures were not uncommon, and the majority of them were caused by trauma [38]. Hence, in our series, although patients obtain good functional outcomes, they are suggested not to undertake vigorous exercise. Besides, comparing to the hollow stem, the blunt-tip-stems in our series behave better in dispersing stress, which benefits preventing further periprosthetic fracture [32]. Moreover, in skeletally immature patients receiving a press-fit fixation, a pre-wire–tied cortex is deemed more tolerant for periprosthetic fracture. To eliminate endoprosthesis fracture, the stem strength was taken into consideration during our design of the stem. Owing to the relatively low mechanical strength of the porous structure, fatigue failure under long-term alternating stress might happen. Therefore, we preserved the inner structure as a solid stem to be covered by a 2.5-mm-thick porous structure layer. Besides, the tunnels for cross-pins were designed as eccentric to the central axis of the stem to avoid occupying the majority of the solid stem which might jeopardize the stem strength. Consequently, no endoprosthesis fracture was observed in our follow-up. Finally, benefitting from the preservation of peri-knee epiphysis, the leg length discrepancy is not severe. The only two patients who encountered leg length discrepancy are considered resulting from the resection of the proximal femoral epiphysis and distal tibial epiphysis.

The median MSTS-93 score of all patients was 26 (range, 23 to 30). Reasonable preservation of the native joints at two ends of the femur or tibia benefits the restoration of lower-limb function [36]. In the literature, the patients with segmental bone defects in the lower extremities received an MSTS score ranging from 22.5 to 28 [29, 31, 35–38]. However, the majority of these endoprosthetic reconstructions involved diaphysis with the assistance of cement, and satisfactory function was obtained resulting from satisfactory initial stability and following early mobilization. In patients receiving uncemented fixation to a critical stump, to counterbalance early rehabilitation for better function and prolonged immobilization for bone healing is still challenging. In our series, personalized rehabilitation programs were applied, and our patients with intercalary reconstruction received similar functional restoration with a median MSTS score of 27.5 (femur) and 26 (tibia). Meanwhile, the patients received hip hemiarthroplasty, and proximal femoral endoprosthesis replacement obtained acceptable functional outcome with a median MSTS score of 24.5. The result is comparable to the literature with an MSTS score range of 18 to 24 [57–61].

## Conclusions

A 3D-printed custom-made ultra-short stem with a porous structure provides acceptable early outcomes in patients who received peri-knee metaphyseal reconstruction. With detailed preoperative design and precise intraoperative techniques, the reasonable initial stability benefits osseointegration to osteoconductive porous titanium, and therefore ensures short- and possibly long-term durability. Personalized adaptive endoprosthesis, careful intraoperative operation, and strict follow-up management enable effective prevention and treatment of complications. The functional results in our series were acceptable thanks to reliable fixation in the bone-endoprosthesis interface and an individualized rehabilitation program. These positive results indicate this device series can be a feasible alternative in critical bone defect reconstruction. Nevertheless, longer follow-up is required to determine whether this technique is superior to other forms of fixation.

## Data Availability

The data that support the findings of this study are available from the corresponding author upon reasonable request.

## Abbreviations

3D: Three-dimensional
MSTS: Musculoskeletal Tumor Society
CT: Computerized tomography
MRI: Magnetic resonance imaging
